# The Role of CD4^+^ T Cells and Microbiota in the Pathogenesis of Asthma

**DOI:** 10.3390/ijms222111822

**Published:** 2021-10-31

**Authors:** Jiung Jeong, Heung Kyu Lee

**Affiliations:** Graduate School of Medical Science and Engineering, Korea Advanced Institute of Science and Technology (KAIST), Daejeon 34141, Korea; skjung1371@kaist.ac.kr

**Keywords:** asthma, T cell, eosinophil, neutrophil, microbiota, commensal, dysbiosis

## Abstract

Asthma, a chronic respiratory disease involving variable airflow limitations, exhibits two phenotypes: eosinophilic and neutrophilic. The asthma phenotype must be considered because the prognosis and drug responsiveness of eosinophilic and neutrophilic asthma differ. CD4^+^ T cells are the main determinant of asthma phenotype. Th2, Th9 and Tfh cells mediate the development of eosinophilic asthma, whereas Th1 and Th17 cells mediate the development of neutrophilic asthma. Elucidating the biological roles of CD4^+^ T cells is thus essential for developing effective asthma treatments and predicting a patient’s prognosis. Commensal bacteria also play a key role in the pathogenesis of asthma. Beneficial bacteria within the host act to suppress asthma, whereas harmful bacteria exacerbate asthma. Recent literature indicates that imbalances between beneficial and harmful bacteria affect the differentiation of CD4^+^ T cells, leading to the development of asthma. Correcting bacterial imbalances using probiotics reportedly improves asthma symptoms. In this review, we investigate the effects of crosstalk between the microbiota and CD4^+^ T cells on the development of asthma.

## 1. Introduction

Asthma is a common respiratory disease involving chronic airway inflammation, primarily caused by allergens such as house dust mites (HDMs), pollen, and animal dander [1]. In general, the prevalence of asthma is approximately 15–20%, but this varies by country [2]. Chronic inflammation resulting from continuous inhalation of allergens can lead to airway remodeling, which in turn can induce various symptoms associated with asthma, such as cough, dyspnea, and wheezing due to airway narrowing [1].

Steroids are often prescribed to control airway inflammation and represent the gold standard for asthma treatment [3]. Although steroid use has improved the quality of life of many asthma patients [4], some patients with severe asthma are refractory to current steroid treatment protocols [3]. These severe asthma patients have poorer quality of life due to a higher frequency of asthma attacks [5]. A variety of drugs for treating severe asthma have been developed in recent years, including mepolizumab, reslizumab, benralizumab and dupilumab [6]. However, these drugs were developed for patients with T helper (Th)2 asthma, and unfortunately, no drugs for patients with non–Th2-asthma are currently available. Thus, novel therapeutic targets for drugs to treat non–Th2-asthma are needed, but the development of such drugs will require elucidation of the mechanism underlying the role of CD4^+^ T cells in asthma pathogenesis.

Two asthma phenotypes have been described, Th2 and non-Th2, which are determined by CD4^+^ T cells [7]. The asthma phenotype can change depending on which type of CD4 T cell is differentiated; consequently, the response to asthma drugs can change accordingly [8]. Th2-asthma (i.e., eosinophilic asthma) is characterized by eosinophilic infiltrate in the sputum [7]. The pathogenesis of eosinophilic asthma is characterized by secretion of high levels of interleukin (IL)-4, IL-5 and IL-13 by Th2 cells [1]. In general, eosinophilic asthma is responsive to steroid treatment, and severe eosinophilic asthma is effectively treated by various newly developed drugs [5]. Non-Th2 asthma (i.e., neutrophilic asthma), by contrast, is characterized by neutrophilic infiltrate in the sputum [7] and secretion of high levels of interferon gamma (IFN-γ) and IL-17 by Th1 and Th17 cells. In contrast to Th2-asthma, non-Th2 asthma does not respond steroids or newly developed asthma drugs [7]. As the disease progression pattern and asthma treatment options differ depending on the differentiation of CD4^+^ T cells, elucidating the biological roles of CD4^+^ T cells in the pathogenesis of asthma is critical for developing effective asthma treatments and predicting patient prognosis.

Although CD4^+^ T cells and other immune cells play key roles in the pathogenesis of asthma, several studies have reported a relationship between the host microbiota and asthma [9,10,11]. Commensal bacteria, which constitute a subtype of the microbiota, are symbiotic bacteria [12]. An adult male weighing 70 kg reportedly harbors approximately 3.8 × 10^13^ commensal bacteria [13]. Approximately 29% of commensal bacteria reside in the gastrointestinal tract, 26% in the oral cavity, 21% on the skin, 14% in the airways, 9% in the urogenital tract, and 1% in the blood [12]. Commensal bacteria perform a variety of biological functions important to the host, including fermentation of undigested dietary carbohydrates, synthesis of bile acids and vitamins, and immune surveillance [14]. Importantly, alterations in the composition of commensal bacteria have been associated with various chronic inflammatory diseases, such as asthma, inflammatory bowel disease, and obesity [15]. Recent literature indicates that the composition of beneficial and harmful bacteria in the host determines the disease pattern of asthma [16]. The same study revealed that various environmental factors that affect these bacteria also affect the differentiation of CD4^+^ T cells, resulting in the development of asthma [16].

In this review, we discuss the detailed mechanism of the pathogenesis of asthma as it relates to Th2-asthma and non-Th2 asthma, with a particular focus on CD4^+^ T cells. In addition, we discuss the role of the bacterial microbiota in the induction of asthma and its effect on CD4^+^ T cells in asthma.

## 2. Th2-Asthma with Eosinophilic Inflammation

**Figure 1:**
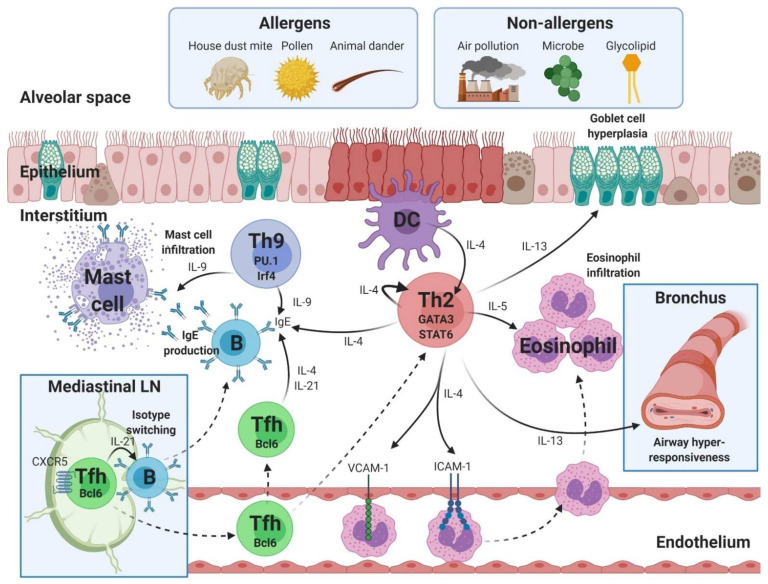
Pathogenesis of eosinophilic asthma mediated by T helper (Th)2, Th9 and T follicular helper (Tfh) cells. The development of eosinophilic asthma is associated with the Th2, Th9 and Tfh subtypes of CD4^+^ T cells. Th2 cells play roles in eosinophilic infiltration, goblet cell hyperplasia, airway hyperresponsiveness, immunoglobulin (Ig)E production, and upregulation of endothelial molecules, including vascular cell adhesion molecule (VCAM)-1 and intercellular adhesion molecule (ICAM)-1. GATA-binding protein 3 (GATA3) and signal transducer and activator of transcription (STAT)6 are transcriptional factors in Th2 cells. Th9 cells mediate mast cell infiltration and IgE production. PU and Irf4 are transcriptional factors in Th9 cells. Bcl6-expressing Tfh cells mediate isotype switching and IgE production. Text color: Black, cytokine. LN, lymph node; DC, dendritic cell; IL, interleukin; Irf4, interferon regulatory factor 4; CXCR5, C-X-C chemokine receptor type 5. Figure created using BioRender.com (accessed on 6 September 2021).

Th2 cells play a central role in the development of Th2-asthma [17]. The hallmark of Th2-asthma is infiltration of the airways by eosinophils. Eosinophilic asthma is diagnosed when the proportion of eosinophils in the sputum is >3% [17]. Th2-asthma can be caused by allergens and non-allergens, including pollutants, microbes, and glycolipids [18]. Approximately 50% of asthmatic adults have Th2-asthma [5]. Although various immune cells are involved in the pathogenesis of Th2-asthma, the Th2, Th9, and T follicular helper cell (Tfh) CD4^+^ T cell subtypes play particularly key roles (Figure 1).

### 2.1. Th2 Cells

Th2 cells constitute a subtype of CD4^+^ T cells [19]. Th2 differentiate in response to IL-4 secreted by dendritic cells (DCs), and innate lymphoid cell group 2 (ILC2) promotes the expression of master transcription factors such as GATA-binding protein 3 (GATA3) and signal transducer and activator of transcription (STAT)6 [19]. Differentiated Th2 cells defend the host against extracellular parasites and secrete various Th2 cytokines, including IL-4, IL-5 and IL-13 [20].

Th2 cytokines play a major role in airway eosinophilic infiltration in Th2-asthma [21]. Compared with healthy control subjects, expression of the Th2 cytokine-related genes *IL-5*, *GPR55*, and *ELAVL1* is upregulated in peripheral blood mononuclear cells (PBMCs) of asthma patients [22].

IL-4 secreted by Th2 cells binds to the IL-4 receptor (IL-4R) in an autocrine manner to continuously initiate Th2 differentiation [23]. Th2-derived IL-4 also promotes allergen-specific immunoglobulin (Ig)E class switching in B cells [24], and upregulates the expression of intercellular adhesion molecule-1 and vascular cell adhesion molecule (VCAM)-1 in endothelial cells in the lungs, resulting in eosinophil recruitment [24].

IL-5 also plays a critical role in eosinophilic inflammation [25]. Foster et al. reported that IL-5–deficient mice exhibit reduced airway eosinophilia despite allergen-induced allergic inflammation [26]. In the bone marrow, IL-5 promotes the differentiation of myeloid precursor cells to mature eosinophils [27]. Circulating mature eosinophils that were triggered to differentiate by IL-5 then adhere to VCAM-1 on endothelial cells and migrate to the bronchial lumen [28]. Accumulation of mature eosinophils in the bronchial lumen exacerbates eosinophilic asthma because activation of Jak2 and Raf-1 inhibits eosinophil apoptosis [25]. In addition, eosinophil survival is prolonged due to the upregulation of mitogen-activated protein kinase genes [25].

IL-13 plays an important role in airway remodeling [29]. IL-13–STAT6 signaling in human epithelial cells induces goblet cell hyperplasia via the upregulation of the mucin 5AC gene [30]. In addition, IL-13 promotes airway hyperresponsiveness, which is aggravated narrowing of the airways in response to external stimuli, by upregulating smooth muscle cell contractility and pulmonary fibrosis [31].

As Th2 cytokines have a marked effect on the occurrence of eosinophilic asthma, various therapeutic agents targeting Th2 cytokines have been developed [6]. Current Th2 cytokine–targeted therapies approved by the Food and Drug Administration can be divided into two classes: drugs that target cytokines (e.g., mepolizumab and reslizumab), and drugs that target cytokine-binding receptors (e.g., benralizumab and dupilumab).

Mepolizumab, an IgG1 monoclonal antibody targeting IL-5, is administered via subcutaneous injection of 100 mg every 4 weeks [6]. Compared with the placebo, mepolizumab reduced glucocorticoid and asthma exacerbation in patients with eosinophilic asthma [32,33]. Reslizumab, an IgG4 monoclonal antibody targeting IL-5, is administered via intravenous injection of 3 mg/kg every 4 weeks [6]. Reslizumab also reduces the number of acute exacerbations and the amount of maintenance steroids required in patients with moderate to severe eosinophilic asthma [34].

Benralizumab, a humanized IgG1 monoclonal antibody targeting IL-5 receptor α, is administered via subcutaneous injection of 30 mg every 8 weeks [6]. Compared with the placebo, benralizumab decreased glucocorticoid use by 75% and decreased the number of asthma exacerbations by 70% in patients with severe eosinophilic asthma [35]. Dupilumab, an IL-4Rα antagonist, is administered via subcutaneous injection every 2 weeks [36]. Compared with the placebo, dupilumab decreased the number of asthma exacerbations by 47.7% in patients with moderate to severe uncontrolled asthma [36]. Furthermore, dupilumab improves lung function, which has not been demonstrated with the other Th2 cytokine–targeted therapies [36]. After 12 weeks of dupilumab use, an improvement in forced expiratory volume in 1 s (FEV1) was observed, with an average increase in FEV1 of 0.32 L [36].

In addition to these cytokines and cytokine-binding receptor-targeted therapy, drugs targeting Th2 transcription factors are also under development [37]. For example, SB010, a GATA3-specific DNAzyme that inhibits transcription of the GATA3 gene, improved lung function and decreased plasma IL-5 levels compared with the placebo [38]. However, that study had several limitations, such as the small study group involving only 40 asthma patients [38]. Large-scale studies of SB010 targeting patients with severe eosinophilic asthma are thus needed.

### 2.2. Th9 Cells

Recent reports suggest that Th9 cells induce allergic reactions and inflammatory responses [39]. Th9 cells are a subset of CD4^+^ T cells that secrete IL-9 and were initially thought to be a subtype of Th2 cells [40]. However, research has revealed that Th9 cells do not produce IL-4, Il-5, or IL-13 and only secrete IL-9 [41]. In addition, Th9 cells express PU.1 and interferon regulatory factor 4 (Irf4) as transcription factors [42]. Th9 cells are therefore recognized as a new subtype of CD4^+^ T cells due to differences compared with conventional Th2 cells in terms of the cytokines and transcription factors produced [41].

Th9-derived IL-9 plays an important role in the development of eosinophilic asthma by assisting the action of Th2 cells [21]. For example, IL-9 enhances IgE production by B cells in conjunction with Th2-derived IL-4. Petit-Frere et al. reported that simultaneous administration of IL-4 and IL-9 exhibited synergistic effects that resulted in upregulation of IgE production [43]. McLane et al. reported that serum IgE levels were elevated in IL-9 transgenic mice compared with normal mice [44]. Analyses of PBMCs isolated from patients with allergen-induced asthma revealed a positive correlation between the number of Th9 cells and plasma IgE level [45]. Other studies found that IL-9 exacerbates eosinophilic inflammation by amplifying the effects of Th2 cytokines. Temann et al. found that compared with normal mice, transgenic mice overexpressing IL-9 exhibited increased production of Th2 cytokines, including IL-5 and IL-13 [46]. The increased levels of IL-5 and IL-13 resulting from IL-9 stimulation increase eosinopoiesis in the bone marrow and enhance goblet cell metaplasia of epithelial cells [47,48]. Chang et al. reported that mice with T cell-specific deletion of PU.1 exhibit reduced OVA-induced eosinophilic inflammation compared with wild-type mice [49].

A unique role of IL-9 compared with Th2 cytokines is the effect on the infiltration of mast cells in lungs. It was previously thought that Th2 cytokines, including IL-4 and IL-13, were responsible for mastocytosis [50]. However, Sehra et al. demonstrated that IL-9 derived from Th9 cells regulates mast cell infiltration in the lungs [51]. Using adoptive Th9 transfer, they found that only IL-9 blockade—and not IL-13 blockade—effectively reduced the infiltration of mast cells in the lungs [51].

Several murine studies examining IL-9 blockade demonstrated effective improvement in eosinophilic asthma factors such as inflammation, suggesting that IL-9 is a novel therapeutic target for treating eosinophilic asthma [52,53]. Unfortunately, however, a randomized controlled trial involving over 300 asthma patients did not find any beneficial improvement in asthma symptoms and lung function compared with the placebo group in patients treated with MEDI-528, a humanized IgG1 monoclonal antibody that inhibits the function of IL-9 [54]. JQ1, a bromodomain-containing protein 4 inhibitor that suppresses chromatin looping, resulting in reduced IL-9 transcription, has attracted recent attention for its potential in Th9 cell-targeted therapies [55]. In a murine study performed by Xiao et al., JQ1 alleviated OVA-induced allergic inflammation [56]. However, the short half-life of JQ1 currently poses an obstacle to clinical use [57]. Therefore, it will be necessary to develop improved Th9 cell-targeted drugs that can be used in asthma patients.

### 2.3. Tfh Cells

Tfh cells constitute a subset of CD4^+^ T cells that localize primarily in lymphoid tissues and function as key regulators of B-cell functions, including proliferation, cytokine production, and isotype switching [58]. When DCs secrete IL-6 in lymphoid tissues after allergen binding, naïve CD4^+^ T cells differentiate into C-X-C chemokine receptor type 5 (CXCR5)-expressing Tfh cells [59]. Regulated by the transcription factor B-cell lymphoma 6 (Bcl6), Tfh cells then secrete IL-4 and IL-21 [60].

Tfh-derived cytokines are major stimulators of IgE production by B cells. Previous studies indicated that IL-4 and IL-9 are involved in IgE production [61]. Kobayashi et al. reported reduced levels of serum IgE in T cell-specific Bcl6-depleted mice compared with control mice, despite no changes in levels of Th2 cytokines such as IL-4, IL-5, and IL-13 [62]. Noble and Zhao reported abnormalities in class switching of IgG as well as IgE in T cell-specific IL-6R mutant mice [63]. A study in humans reported a positive correlation between circulating Tfh cells and HDM-specific IgE [64]. These results suggest that Tfh cells—but not Th2 cells—play an important role in IgE production.

Tfh cells also play a role in amplifying the effects of Th2 cytokines during the induction of Th2-asthma. Two hypotheses have been proposed to explain this phenomenon. The first hypothesis holds that peripheral Tfh cells, which do not express CXCR5, migrate directly from the mediastinal lymph nodes to the lungs. The second hypothesis holds that Tfh cells are transformed into pathogenic Th2 cells. Using IL-21–green fluorescent protein reporter mice, Coquet et al. concluded that IL-21–producing cells presumed to be of Tfh origin localize in lungs and amplify Th2 cell responses via the binding of IL-21 to IL-21R on Th2 cells [65]. In contrast, Ballesteros-Tato et al. reported that IL-4–producing Tfh cells can differentiate into precursors of pathogenic Th2 cells [66].

Two types of therapeutics targeting Tfh cells have been developed: an inducible T-cell costimulatory (ICOS) ligand–targeted antibody, and a CXCR5-targeted therapy. Uwadiae et al. reported that the ICOS ligand–targeted antibody alleviated HDM-induced eosinophilic inflammation in a murine model [67]. Using PBMCs isolated from asthma patients and healthy controls, Zhang et al. reported that miR-192, a small, non-coding RNA that regulates CXCR5 expression, inhibits the function of Tfh cells [68]. Because Tfh cell-targeted therapies are still in the experimental stage, clinical trials of the ICOS ligand–targeted antibody and miR-192 are in progress.

## 3. Non-Th2 Asthma with Neutrophilic Inflammation

**Figure 2:**
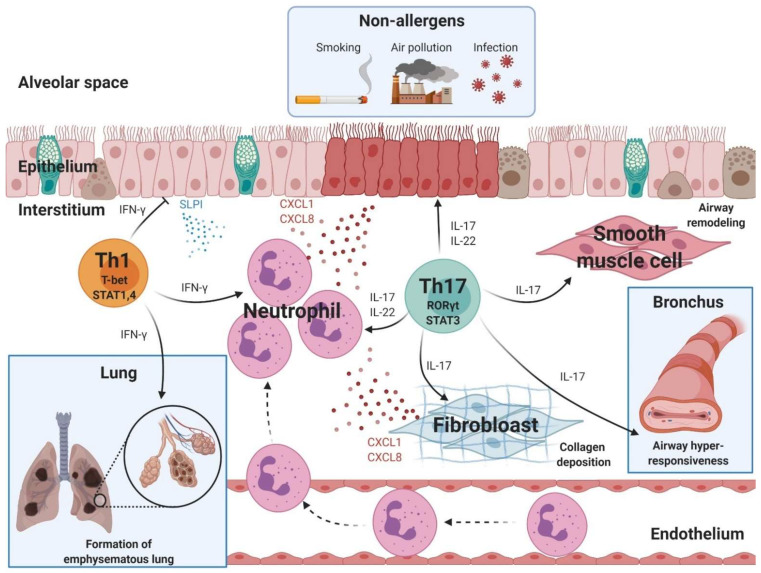
Pathogenesis of neutrophilic asthma mediated by Th1 and Th17 cells. The development of neutrophilic asthma is associated with subtypes of CD4^+^ T cells including Th1 and Th17 cells. Th1 cells are involved in mediating the infiltration of neutrophils and the formation of emphysematous lung. T-bet, STAT1, and STAT4 are transcriptional factors in Th1 cells. Th17 cells play critical roles in neutrophil infiltration, airway remodeling, collagen deposition, and airway hyperresponsiveness. RORγt and STAT3 are transcriptional factors in Th17 cells. Text color: Black, cytokine; Red, chemokine; Blue, anti-protease. T-bet, T-box protein expressed in T cells; RORγt, RAR-related orphan receptor gamma; CXCL, C-X-C motif chemokine ligand; SLPI, secretory leukocyte peptidase inhibitor. The figure was created using BioRender.com (accessed on 6 September 2021).

Non–Th2 asthma refers to asthma involving <3% eosinophilic infiltration in the sputum [7]. Fewer than 50% of asthma patients are diagnosed with non–Th2 asthma, which primarily occurs in adulthood [69]. Non–Th2 asthma is induced by non-allergenic factors such as smoking, air pollution, inhaled ozone, and infection [7]. Patients with non–Th2 asthma suffer from poor asthma control and experience frequent exacerbations of asthma symptoms due to the development of medication resistance [70]. Neutrophil infiltration is a key characteristic of patients presenting with non–Th2-asthma. Among the CD4^+^ T cell subsets, Th17 and Th1 cells reportedly play important roles in neutrophil infiltration of the airways (Figure 2) [7].

### 3.1. Th17 Cells

Th17 cells exert a significant effect on neutrophilic inflammation during the development of asthma [7]. Th17 cells secrete IL-17A, IL-17F, and IL-22 as part of the response against extracellular pathogens and fungi. In addition, Th17 cells express the transcription factors STAT3, RAR-related orphan receptor gamma (RORγt), and RORα [20]. To differentiate Th17 cells, IL-6, IL-23, and TGF-β are required [19].

Th17 cytokines such as IL-17A, IL-17F, and IL-22 promote neutrophil recruitment in the airways. Studies in human cell lines reported that exposure to IL-17 enhances the secretion of neutrophil chemotaxis factors such as C-X-C motif chemokine ligand (CXCL)1 and CXCL8 by stimulating epithelial cells and fibrocytes [71,72,73]. Newcomb et al. found reduced neutrophil infiltration in the airways of IL-17A–knockout mice [74]. Camargo et al. reported that blockade of IL-17 reduces lipopolysaccharide-induced neutrophilic inflammation in the airways of mice [75].

Th17 cytokines are also involved in airway remodeling and hyperresponsiveness via binding to IL-17RA and IL-17RC on airway smooth muscle cells [76,77,78]. In an animal model study of airway remodeling, Ramakrishnan et al. demonstrated that IL-17-induces autophagy in fibroblasts, which initiates mitochondrial dysfunction that results in collagen deposition [79]. In a study examining hyperresponsiveness, Chiba et al. reported that the complex formed by the binding of IL-17A to the IL-17R on smooth muscle cells stimulates increased production of RhoA protein, which plays a role in upregulating intracellular calcium concentrations, resulting in enhanced smooth muscle cell contractility [80]. These data from murine studies suggest that antibodies targeting IL-17A would reduce airway remodeling and airway hyperresponsiveness [75,81].

Several other studies have reported a link between steroid resistance and Th17 [82,83]. Two hypotheses have been proposed to explain this possible relationship. The first hypothesis involves the steroid resistance of Th17 cells, whereas Th2 cells are sensitive to steroids. The second hypothesis suggests that steroids promote Th17 cell differentiation. Nanzer et al. examined PBMCs of asthma patients and showed that steroids did not inhibit cytokine synthesis by Th17 cells, in contrast to PBMCs of healthy controls [82]. However, Chambers et al. reported that steroid dose-dependent Th17 cytokine synthesis plays a role in in vitro activation of human PBMCs [83]. These data explain the high proportion of Th17 cells in asthma patients with steroid resistance.

Unfortunately, antibody-based therapy targeting IL-17A did not improve asthma symptoms in clinical trials [84]. However, treatment of a patient with chronic psoriasis and asthma with ustekinumab, a humanized IgG1 monoclonal antibody targeting both IL-12 and IL-23, resulted in improvement in asthma symptoms and a reduction in asthma maintenance medication [85]. Collectively, the above results suggest that alleviating Th17-related asthma requires the control of not just one Th17 cytokine pathway but all pathways that simultaneously regulate Th17 cytokines.

### 3.2. Th1 Cells

Th1 cells are also major inducers of neutrophilic inflammation in non–Th2-asthma. Th1 cells function in protecting host tissues against intracellular bacteria and viruses [20]. Th1 transcription factors include STAT1, STAT4, and T-bet (T-box protein expressed in T cells) [86]. Th1 cells, which differentiate in response to IL-12, secrete IFN-γ [20].

According to the hygiene hypothesis, Th1 cells inhibit the development of eosinophilic asthma, whereas Th2 cells promote the development of eosinophilic asthma [87]. However, recent studies reported that Th1 cells play an important role in the pathogenesis of severe non–Th2-asthma. Cui et al. reported that administration of OVA-specific Th1 aggravated neutrophilic inflammation in the lungs [88]. Raundhal et al. reported increased levels of the Th1 cytokine IFN-γ in bronchoalveolar lavage fluid of non–Th2-asthma patients [89]. Additionally, increased neutrophilic infiltration and IFN-γ mRNA expression in the sputum were observed in patients with severe asthma compared with patients with mild to moderate asthma [90]. These data suggest that Th1 cells play a role in the pathogenesis of severe non–Th2-asthma.

Th1 cell-derived IFN-γ is associated with airway hyperresponsiveness and pathologic changes in the lungs. Raundhal et al. found that IFN-γ reduces the expression of secretory leukocyte peptidase inhibitor, which neutralizes proteases in epithelial cells, thus aggravating airway hyperresponsiveness [89]. IFN-γ transgenic mice expressing high levels of IFN-γ developed emphysematous lungs, which is frequently observed in asthma–chronic obstructive pulmonary disease (COPD) overlap [91]. In the future, it will be necessary to develop new asthma treatments targeting Th1 cells.

## 4. Beneficial and Harmful Bacteria in the Pathogenesis of Asthma

Many species of bacteria live in symbiosis with hosts and play an important role in the development of asthma [92]. Beneficial species of bacteria suppress asthma, whereas harmful bacteria induce asthma [93]. In this section, we summarize the roles of these two types of bacteria in the pathogenesis of asthma.

### 4.1. Beneficial Bacteria with Anti-Asthmatic Effects

Beneficial bacteria include symbiotic species of the genera *Lactobacillus*, *Bifidobacterium*, *Lachnospira* and *Akkermansia*. Fermented foods such as yogurt and kimchi contain numerous beneficial bacteria [94,95]. Recently, probiotic products incorporating these beneficial bacteria have been used to reduce the risk of asthma [16].

Members of the genus *Lactobacillus* are gram-positive anaerobic bacteria that play a protective role in the pathogenesis of asthma. Spacova et al. reported that intranasal administration of *Lactobacillus rhamnosus* alleviated pollen-induced eosinophilic inflammation in the lungs [96]. According to Li et al., butyrate, a short-chain fatty acid (SCFA) generated from the fermentation of fiber by *L. reuteri*, exhibits anti-inflammatory activity in patients with asthma [97]. In a randomized, placebo-controlled study, the asthma patients group who received *L. gasseri* A5 daily for 2 months exhibited higher lung function scores (peak expiratory flow rate) and lower clinical symptom scores, indicating improvement in asthma compared with patients who received the placebo [98].

Members of the genus *Bifidobacterium* are Gram-positive anaerobic bacteria that exert immunomodulatory effects that suppress the development of asthma. In a study by Raftis et al., *Bifidobacterium breve* strain MRx0004 suppressed HDM-induced inflammation and the number of eosinophils and neutrophils [99]. Administration of *Bifidobacterium* upregulates IL-10–producing regulatory T cells (Tregs), a type of CD4^+^ T cell that suppresses hyper-activation of immune responses [100]. In a randomized controlled study of pediatric asthma patients, administration of a *Bifidobacterium* mixture resulted in improvement in clinical symptoms and quality of life compared with patients who received the placebo [101].

Members of the genus *Lachnospira* are gram-positive anaerobic bacteria that function as major producers of SCFAs such as acetate, propionate, and butyrate [102]. These SCFAs bind to G-protein–coupled receptor (GPR) 43 on the surface of naïve CD4^+^ T cells [103]. The SCFA-GPR43 complex, in turn, promotes acetylation of the Treg transcription factor Foxp3 by suppressing histone deacetylase (HDAC) in naïve CD4^+^ T cells [104]. Arrieta et al. found that fecal transplantation with a mixture of *Lachnospira* reduced OVA-induced neutrophilic inflammation to a greater degree than the control [105]. It is possible that increased levels of SCFAs produced by *Lachnospira* enhance Treg differentiation and suppress pathogenic immune cells.

Members of the genus *Akkermansia* are gram-negative anaerobic bacteria that inhibit the development of asthma by promoting differentiation of Treg [106]. In a study by Kuczma et al., *Akkermansia-*derived antigenic peptide-induced anergy of T cells and increased the peripheral Treg population [107]. Michalovich et al. showed that oral administration of *A. muciniphila* reduced OVA-induced eosinophilic inflammation [106]. In a cross-sectional case-controlled study, *A. muciniphila* was decreased in the stool of asthma patients compared to healthy controls [108]. In addition, the fecal concentration of *A. muciniphila* was negatively correlated with asthma severity [106]. These results suggest that *Akkermansia* plays a protective role in the development of asthma.

Other bacteria also reportedly exert beneficial effects in inhibiting the induction of asthma, including members of the genera *Veillonella*, *Faecalibacterium* and *Rothia* [93].

### 4.2. Harmful Bacteria with Pro-Asthmatic Effects

Bacteria that exert harmful effects with respect to asthma include pathogens of the genera *Clostridium*, *Staphylococcus* and *Pseudomonas*. Under certain conditions, these harmful bacteria reportedly exacerbate enterocolitis and pneumonia [109,110]. Additionally, colonization by harmful bacteria reportedly increases the risk of asthma development [16].

Members of the genus *Clostridium* are gram-positive anaerobic bacteria that reportedly aggravate asthma. Nimwegen et al. reported that colonization by *Clostridium difficile* within 1 month after birth is associated with an increased risk of developing childhood asthma [111]. In a pediatric cohort study, asthma patients exhibited higher numbers of *C. neonatale* [112]. Colonization by *Clostridium* species was positively correlated with fecal IgE levels in a childhood asthma study, indicating that the presence of *Clostridium* increases the risk of asthma [113]. Although the detailed mechanism of the role of *Clostridium* in asthma pathogenesis has not been elucidated, infections involving *Clostridium* could cause excessive inflammation and increase the pathologic immune cells, thereby worsening asthma.

Members of the genus *Staphylococcus* are gram-positive bacteria that induce eosinophilic asthma. Stentzel et al. demonstrated that serine protease–like proteins (Spls), extracellular proteases expressed by *Staphylococcus aureus*, exacerbate eosinophilic asthma [114]. Proteases such as Spls bind to the protease-activated receptor-2 on epithelial cells, which then secrete alarmins such as IL-33 and TSLP, which in turn activate ILC2 and induce a Th2 response [115]. According to the National Health and Nutrition Examination Survey (NHANES), nasal colonization by *S. aureus* is associated with increased severity of asthma symptoms [116].

Members of the genus *Pseudomonas* are gram-negative bacteria known as opportunistic pathogens that cause respiratory diseases such as asthma, COPD, and bronchiectasis. *Pseudomonas aeruginosa* is the second most common bacteria in sputum cultures of patients with severe asthma [117]. According to Tuli et al., planktonic exo-proteins isolated from *P. aeruginosa* damage the mucosal barrier, thereby exacerbating asthma and chronic rhinosinusitis [118]. Flagellin isolated from *P. aeruginosa* was shown to increase secretion of the potent neutrophil chemoattractants IL-6 and IL-8 in human epithelial cells [119]. In a human study conducted by Green et al., asthma patients in which *P. aeruginosa* was the dominant pathogenic bacteria exhibited more severe neutrophilic inflammation and steroid resistance than patients in which other species were dominant [120].

In addition, nasopharyngeal colonization by members of the genera *Streptococcus*, *Moraxella*, and *Haemophilus* within the first year of life is associated with an increased risk of childhood asthma [121].

## 5. Dysbiosis-Induced Asthma

**Figure 3:**
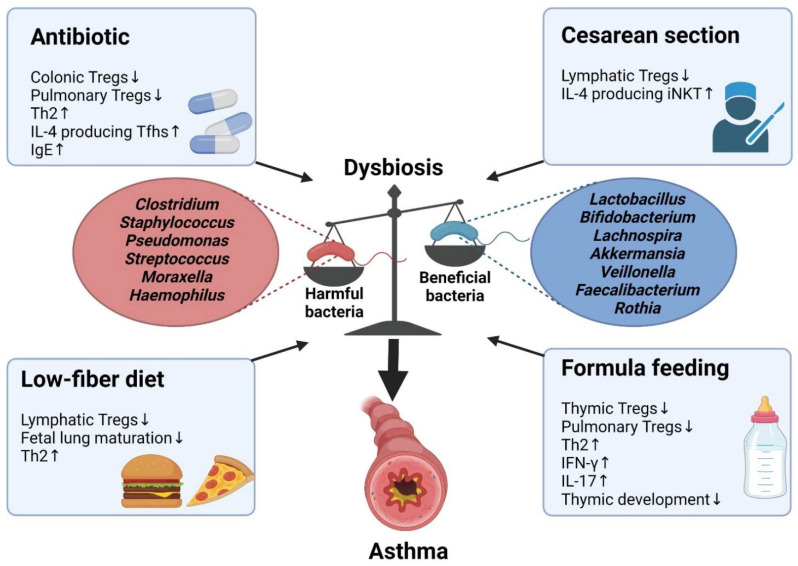
Development of dysbiosis-induced asthma. Beneficial bacteria suppress asthma, whereas harmful bacteria induce asthma. Dysbiosis can be caused by many factors, such as antibiotic use, cesarean section, low-fiber diet, and formula feeding. Dysbiosis influences the differentiation of T cells, resulting in asthma development. Tregs, regulatory T cells; Tfhs, T follicular helper cells; IgE, immunoglobulin E; iNKT, invariant natural killer T; IFN-γ, interferon gamma. The figure was created using BioRender.com (accessed on 6 September 2021).

Dysbiosis is a disruption of the immune system caused by a dysregulation of microbiota homeostasis [122]. Several factors can initiate dysbiosis, including the use of antibiotics in the prenatal or neonatal periods, cesarean section, consumption of a low-fiber diet by the mother, or formula feeding [123]. Dysbiosis reportedly aggravates asthma by decreasing the number of Tregs and increasing the numbers of pathologic Th2 and Th17 cells [108,124]. In this section, we discuss how alterations in CD4^+^ T cells during dysbiosis affect the pathogenesis of asthma (Figure 3).

### 5.1. Antibiotics

Several reports have indicated that antibiotic use can induce asthma [125,126,127]. The use of antibiotics before and after pregnancy reportedly increases the incidence of childhood asthma [128]. The functions of CD4^+^ T cells can be altered by antibiotic use, subsequently provoking the development of eosinophilic asthma. Murine studies demonstrated that antibiotic-induced dysbiosis exacerbates Th2-driven allergic inflammation by reducing numbers of Tregs in the colon [129] and lungs [129,130]. Hong et al. reported abnormal immune responses to undigested food in antibiotic-treated mice, resulting in increases in food antigen-driven IL-4–producing Tfhs and IgE production [131]. In a prospective cohort study, infants who received antibiotics between birth and 1 year of age had a 50% increased risk of childhood asthma [127].

### 5.2. Cesarean Section

Children delivered by cesarean section are reportedly at increased risk of asthma. According to Shao et al., delivery mode is the most influential factor in the formation of the neonatal gut microbiota [132]. Babies born via vaginal delivery obtain commensal bacteria from the mother’s vagina, whereas babies born via cesarean section receive commensal bacteria from the mother’s skin [133]. Kim et al. reported that infants born via cesarean section harbor fewer asthma-suppressing *Bifidobacterium*, *Lactobacillus*, and *Lachnospira* and more asthma-promoting *Pseudomonas* in the gut [134]. In a murine study conducted by Zachariassen et al., mice born via cesarean section had fewer Tregs and increased numbers of IL-4–producing invariant natural killer T cells in mesenteric lymph nodes [135]. As a result, cesarean section–induced dysbiosis increases the risk of childhood asthma by 3-fold [136].

### 5.3. Low-Fiber Diet

A high-fiber diet protects against asthma. Fiber, a component of plants, is a complex carbohydrate structure composed of β-glycoside–linked glucose monomers [137]. Plant fibers are degraded into SCFAs, including acetate, via fermentation by gut bacteria [138]. Thorburn et al. reported that pregnant mother mice fed a high-fiber diet exhibited increased acetate production, which in turn increased the number of Tregs via HDAC9 inhibition; this led to alleviation of HDM-induced eosinophilic inflammation [139]. Fetal mice provided increased acetate via the placenta exhibited asthma-resistant lung maturation [139]. On the other hand, a low-fiber diet increased Th2 differentiation which led to eosinophilic airway inflammation [140]. Trompette et al. showed that reduction of SCFA by low-fiber diet affected hematopoiesis and increased Th2 cell response [140]. Using data from the 2007–2012 NHANES, Saeed et al. showed that low fiber intake is associated with a higher incidence of asthma as compared with high fiber intake [141].

### 5.4. Formula Feeding

Breast milk contains a variety of components that suppress the development of asthma in children. Mosconi et al. reported that allergen-specific IgG contained in breast milk binds to the Fc receptor of intestinal epithelial cells of the fetus, resulting in allergen-specific Treg induction and reduction of Th2 response [142]. Other breast milk components, including IL-7, cortisol, and microRNAs, aid in thymus development [143]. Ultrasound analyses comparing the size of the thymus of breastfed infants with that of formula-fed infants, the thymus size was reduced by >50% in 4-month-old formula-fed infants [144]. A murine study conducted by Nakajima et al. showed that SCFAs contained in breast milk bind to GPR41 in the fetal thymus and enhance Treg differentiation in both the thymus and peripheral organs [145]. Analyses of PBMCs from formula-fed and breastfed babies showed that formula feeding leads to a reduction in the number of Tregs, resulting in increased levels of pro-inflammatory cytokines such as IFN-γ and IL-17 [146]. A cross-sectional study including 31,049 children reported that formula-fed children had a higher incidence of asthma than breastfed children [147].

## 6. Conclusions

Asthma is a heterogenous disease that can be largely classified as either eosinophilic asthma or neutrophilic asthma. CD4^+^ T cells play important roles in determining the asthma phenotype. Th2, Th9, and Tfh cells are involved in the development of eosinophilic asthma, whereas Th1 and Th17 cells are involved in the development of neutrophilic asthma. Proper classification of the asthma phenotype based on CD4^+^ T cells is essential to determine the optimal asthma treatment and accurately predict the prognosis.

Crosstalk between the microbiota and host immune system is another important factor in asthma development. Beneficial bacteria play a protective role in the pathogenesis of asthma, whereas harmful bacteria exacerbate asthma symptoms. Dysbiosis caused by an imbalance in the microbiota homeostasis alters the differentiation of CD4^+^ T cells, resulting in asthma aggravation. Dysbiosis can be corrected using various probiotic products that were developed to improve asthma symptoms [148,149]. However, these probiotics still play an adjuvant role in the treatment of asthma. To evaluate the microbiota as a potential therapeutic target in greater detail, a precise mechanistic study will be necessary to fully elucidate the effects of the microbiota and CD4^+^ T cells on the pathogenesis of asthma.

## Data Availability

Not applicable.
